# Teaching Multiple Inverse Reinforcement Learners

**DOI:** 10.3389/frai.2021.625183

**Published:** 2021-09-16

**Authors:** Francisco S. Melo, Manuel Lopes

**Affiliations:** INESC-ID, Instituto Superior Técnico, University of Lisbon, Lisbon, Portugal

**Keywords:** optimal teaching, inverse reinforcement learning, heterogeneous multi-agent teaching, class teaching, Markov decision processes

## Abstract

In this paper, we propose the first machine teaching algorithm for multiple inverse reinforcement learners. As our initial contribution, we formalize the problem of optimally teaching a sequential task to a heterogeneous class of learners. We then contribute a theoretical analysis of such problem, identifying conditions under which it is possible to conduct such teaching using the same demonstration for all learners. Our analysis shows that, contrary to other teaching problems, teaching a sequential task to a heterogeneous class of learners with a single demonstration may not be possible, as the differences between individual agents increase. We then contribute two algorithms that address the main difficulties identified by our theoretical analysis. The first algorithm, which we dub SplitTeach, starts by teaching the class as a whole until all students have learned all that they can learn as a group; it then teaches each student individually, ensuring that all students are able to perfectly acquire the target task. The second approach, which we dub JointTeach, selects a single demonstration to be provided to the whole class so that all students learn the target task as well as a single demonstration allows. While SplitTeach ensures optimal teaching at the cost of a bigger teaching effort, JointTeach ensures minimal effort, although the learners are not guaranteed to perfectly recover the target task. We conclude by illustrating our methods in several simulation domains. The simulation results agree with our theoretical findings, showcasing that indeed class teaching is not possible in the presence of heterogeneous students. At the same time, they also illustrate the main properties of our proposed algorithms: in all domains, SplitTeach guarantees perfect teaching and, in terms of teaching effort, is always at least as good as individualized teaching (often better); on the other hand, JointTeach attains minimal teaching effort in all domains, even if sometimes it compromises the teaching performance.

## 1. Introduction

Machines can be used to improve education by providing personalized learning activities. Research on machine teaching and intelligent tutoring systems has proposed different ways by which to attain such personalization (Anderson et al., 1995; Koedinger et al., 1997; Nkambou et al., 2010; Davenport et al., 2012; Patil et al., 2014; Clement et al., 2015). For example, if we consider that a significant part of learning relies on examples, learning efficiency can be greatly improved if the teacher is able to carefully select the examples that are most informative for each particular learner.

However, teaching of human learners is a very challenging problem, due to a number of reasons. First, estimating the cognitive model of a human learner is often a challenge in itself (Corbett and Anderson, 1994; Beck and Xiong, 2013; González-Brenes et al., 2014). Second, it is often convenient to adapt the “level of difficulty” of the teaching contents to the progress of the learner (Lee, 2005). Both assessing the cognitive model of the learner and adapting the teaching contents to her progress require a close interaction between learner and teacher, and a third challenge thus is to ensure that such frequent interactions do not reduce motivation and engagement (Shute, 2011). Finally, one last challenge is finding the best teaching examples at each stage of learning (Rafferty et al., 2011; Clement et al., 2015).

Some of the aforementioned approaches circumvent the need to model the learner by treating her as a “black-box,” i.e., not considering the actual learning process of the learner. That is the case, for example, in most *intelligent tutoring systems* (ITS), which select content as a direct result of the learners' responses during interaction, without explicitly considering the learning process of the particular user. In that way, the contents selected by ITS are tailored to the *observations*, and not tailored to the *learner* (Anderson et al., 1995; Koedinger et al., 1997; Nkambou et al., 2010; Clement et al., 2015; Mota et al., 2015).

*Machine teaching* (MT), on the other hand, considers the problem of finding the smallest set of examples that allows a specific learner to acquire a given concept. MT sets itself apart from standard ITS in that it explicitly considers a specific computational model of the learner (Balbach and Zeugmann, 2009; Zhu, 2013, 2015; Zhu et al., 2018). The optimal amount of training examples needed to teach a target task to a specific learner is known as the *teaching dimension* (TD) of that task-learner pair (Shinohara and Miyano, 1991; Goldman and Kearns, 1995). By optimizing the teaching dimension, machine teaching promises to strongly reduce the effort required from both learner and teacher.

Much like intelligent tutoring systems, machine teaching can be applied in several real-world problems. In this work, we are motivated by examples where we need to teach tasks that are sequential in nature: cognitive tasks such as algebraic computation or algorithms; motor tasks such as industrial maintenance or assembly; etc. We are interested in understanding how such tasks can be efficiently taught to a *heterogeneous class*, i.e., a large number of learners who might have different cognitive and motor skills.

Most MT research so far has focused on single-learner settings in non-sequential tasks—such as Bayesian estimation and classification (Shinohara and Miyano, 1991; Goldman and Kearns, 1995; Balbach and Zeugmann, 2009; Zhu, 2013, 2015; Zhu et al., 2018). Recently, however, some works have considered the extension of the machine teaching paradigm to novel settings. For example:

1. Some works have investigated the impact of group settings on machine teaching results. In the context of non-sequential tasks, Zhu et al. (2017) show that it is possible to teach a heterogeneous class using a common set of examples. The same work also establishes that, by dividing a group of learners in small groups, it is possible to attain a smaller teaching dimension. Yeo et al. (2019) generalize those results for more complex learning problems, and consider additional differences between the learners, e.g., learning rates. Teaching to multiple learners, in the context of classification tasks, has also been considered with more complex learning models, for example when each learner has an exponentially decayed memory (Zhou et al., 2018). Recent works have also considered the case of imperfect labels (Zhou et al., 2020).
  Other approaches that consider multiple learners focus on very different settings. Examples include decomposing a multi-class classification problem into multiple binary classification problems, where the multi-class classifier acts as the “teacher” and the different binary classifiers are the learners (You et al., 2018). Other works also explore the ideas of teaching multiple learners in the context of compressing a complex neural network into multiple simpler networks (Malik et al., 2020).
2. Some works (Walsh and Goschin, 2012; Haug et al., 2018; Melo et al., 2018) investigate the impact that the mismatch between the learner and the teacher's model of the learner may have in the teaching dimension—a situation particularly relevant in group settings. The aforementioned works focus on supervised learning settings, although some more recent works have explored inverse reinforcement learning (IRL) settings (Kamalaruban et al., 2019).
3. Other works have considered machine teaching in sequential decision tasks. Cakmak and Lopes (2012) introduce the first machine teaching algorithm for sequential decision tasks (i.e., when the learners are inverse reinforcement learners). Brown and Niekum (2019) propose an improved algorithm that takes into consideration reward equivalence in terms of the target task representation. The work of Rafferty et al. (2015) considers sequential tasks in a different way; instead of evaluating the quality of learning based on the match between the demonstrated and the learned policy, it infers the understanding of the task by estimating the world model that the learners inferred. Recent approaches for teaching in the context of IRL have considered that interactions are not always possible, providing improvements both for the teacher and learner side (Troussard et al., 2020). Other recent methods have also considered more complex forms of teaching that take into account preferences and constraints (Tschiatschek et al., 2019). In a context of reinforcement learning, rather than IRL, several works have explored these ideas to better understand how humans learn (Chuang et al., 2020), as well as the theoretical teaching dimension of *Q*-learning (Zhang et al., 2020).

From the previous discussion, summarized in Table 1, we see that teaching multiple heterogeneous learners in the context of sequential tasks has not be considered. In this paper, we build on the ideas discussed above and consider the problem of teaching a *sequential task* to a group of *heterogeneous learners* (a “class”). We henceforth refer to a setting where a single teacher interacts with multiple (possibly different) learners as *class teaching*. We follow Cakmak and Lopes (2012) in assuming that the learners are inverse reinforcement learners (Ng et al., 2000), and address the problem of selecting a demonstration that ensures that *all* learners are able to recover a task description that is “compatible” with the target task, in a sense soon to be made precise. Specifically, the paper focuses on the following research question:

*Is it possible to teach a sequential task to a class of heterogeneous inverse reinforcement learners using a single demonstration?*

**Table 1:** Comparative analysis of the most relevant works.

	**Sequential**	**Multiple**	**Uncertainty**
Current paper	×	×	
Brown and Niekum (2019)	×		
Cakmak and Lopes (2012)	×		
Zhou et al. (2020)		×	×
Zhu et al. (2017)		×	
Yeo et al. (2019)		×	×
Melo et al. (2018)			×
Haug et al. (2018)	×		×
Walsh and Goschin (2012)	×		×
Kamalaruban et al. (2019)	×		×

*The column “Sequential” indicates whether the works address sequential tasks; the column “Multiple” indicates whether the different works address multiple learners; finally, the column “Uncertainty” indicates which works consider uncertainty in some form—either in the information provided by the teacher or the teacher's knowledge about the learner. We have also grouped the references along their main focus—the first group is focused on sequential tasks; the second group is focused in settings with multiple learners; the third group is focused on dealing with uncertainty*.

Teaching a sequential task in a class setting, however, poses several additional complications found neither in single-agent settings (Cakmak and Lopes, 2012; Walsh and Goschin, 2012; Haug et al., 2018; Brown and Niekum, 2019), nor on estimation/classification settings (Walsh and Goschin, 2012; Zhu et al., 2017; Yeo et al., 2019). In such setting, we need to teach not only one particular learner but a whole diverse group of learners. The teacher needs to guarantee that all learners learn, while delivering the same “lecture” to everyone. Learner diversity might have different origins, from different learning rates or prior information, to having a completely learning algorithm. Intuitively speaking, we may think that if the differences are large, then each learner needs a particular demonstration and class teaching is not possible. Nevertheless, quantifying what are “large” differences is not trivial.

As an example, in the family of tasks considered by Zhu et al. (2017) or Yeo et al. (2019), learners have large differences in their prior information. But, no matter how large this difference is, all learners can be taught with the same demonstration, even if a larger number of samples is required. In the present work, we investigate what happens in sequential tasks to understand which differences between learners may still allow to teach all of them simultaneously and which differences do not. We discuss the challenges arising when extending machine teaching of sequential tasks to class settings and contribute the first formalization of the problem from the teacher's perspective. We then contribute an analysis of the problem, identifying conditions under which it is possible to teach a heterogeneous class with a common demonstration. From our analysis, we propose two class teaching algorithms for sequential tasks—SplitTeach and JointTeach—and illustrate their performance against other more “naive” alternatives.

In summary, the main contributions of the paper are as follows:

- We contribute the first formalization of the problem of teaching a sequential tasks to a heterogeneous class of inverse reinforcement learners.
- We contribute a theoretical analysis of the aforementioned problem, identifying conditions under which class teaching is possible and is not possible.
- We propose two novel teaching algorithms for sequential tasks—SplitTeach and JointTeach—and discuss their relative merits and inconvenients.
- We illustrate the application of the aforementioned methods in six different simulation class teaching scenarios.

The paper is organized as follows. Section 2 provides an overview of reinforcement learning (RL), IRL, and machine teaching in IRL. Section 3 formalizes the problem of class-teaching a sequential task and provides a theoretical analysis thereof. Section 4 introduces the SplitTeach and JointTeach algorithms, whose performance is then illustrated in section 5. Section 6 concludes the paper.

## 2. Background

In this section, we go over key background concepts upon which our work is built, both to set the nomenclature and the notation. We go over Markov decision problems (MDPs, Puterman, 2005), IRL (Ng et al., 2000), and machine teaching in RL settings (Cakmak and Lopes, 2012; Brown and Niekum, 2019).

### 2.1. Markov Decision Problems

A Markov decision problem (MDP) is a tuple (S,A,P,r,γ), where S is the state space, A is the action space, **P** encodes the transition probabilities, where


$$\begin{array}{l} \text{P}\left(s^{\prime }|s,a\right)=\mathbb{P}\left[S_{t+1}=s^{\prime }|S_{t}=s,A_{t}=a\right], \end{array}$$

and *S*_*t*_ and *A*_*t*_ denote, respectively, the state and action at time step *t*. The function r:S→ℝ is the reward function, where *r*(*s*) is the reward received by the agent upon arriving at a state s∈S. Finally, γ ∈ [0, 1) is a discount factor.

A *policy* is a mapping π:S→Δ(A), where Δ(A) is the set of probability distributions over A. Solving an MDP amounts to computing the optimal policy π^*^ that maximizes the value


$$\begin{array}{l} v^{\pi }\left(s\right)≜𝔼\left[\overset{\infty }{\underset{t=0}{\sum }}\gamma^{t}r\left(s\right)|S_{0}=s,A_{t}\sim \pi \left(\cdot |S_{t}\right)\right] \end{array}$$

for all s∈S. In other words, we have that *v*^π^*^^(*s*) ≥ *v*^π^(*s*) for all policies π and states *s*. We henceforth denote by π^*^(*r*) the optimal policy with respect to the MDP (S,A,P,r,γ), where S, A, **P**, and γ are usually implicit from the context. Writing the value function *v*^π^ as a vector ***v***^π^, we get


(1) $$\begin{array}{l} v^{\pi }=r+\gamma {\text{P}}_{\pi }v^{\pi }={\left(\text{I}-\gamma {\text{P}}_{\pi }\right)}^{-1}r, \end{array}$$

where **P**_π_ is a matrix with component *ss*′ given by:


$${\left[{\text{P}}_{\pi }\right]}_{ss^{\prime }}=\underset{a\in A}{\sum }\pi \left(a|s\right)\text{P}\left(s^{\prime }|s,a\right).$$

### 2.2. Inverse Reinforcement Learning

In IRL, we are provided with a “rewardless MDP” (S,A,P,γ) and a sample of a policy π (or a trajectory obtained by following π) and wish to determine a reward function r^*^ such that π is optimal with respect to r^*^, i.e., π = π^*^(r^*^) for the resulting MDP. If π is optimal, then, given an arbitrary policy π′,


$$\begin{array}{l} r+\gamma {\text{P}}_{\pi }v^{\pi }≽r+\gamma {\text{P}}_{\pi^{\prime }}v^{\pi }, \end{array}$$

where we write ≽ to denote element-wise inequality. Using (1), for a reward *r* to be a valid solution, it must verify the constraint


(2) $$\begin{array}{l} \left({\text{P}}_{\pi }-{\text{P}}_{\pi^{\prime }}\right){\left(\text{I}-\gamma {\text{P}}_{\pi }\right)}^{-1}r≽0. \end{array}$$

Unfortunately, the constraint in (2) is insufficient to identify r^*^. For one, (2) is trivially verified for ***r*** = **0**. More generally, given a policy π, there are multiple reward functions that yield π as the optimal policy. In the context of an IRL problem, we say that two reward functions *r* and *r*′ are *policy-equivalent* if π^*^(*r*) = π^*^(*r*′).^1^ Moreover, the computation of the constraint in (2) requires the learner to access the complete policy π. In practice, however, it is inconvenient to explicitly enunciate π. Instead, the learner may be provided with a *demonstration* consisting of a set


$$\begin{array}{l} D=\left\{\left(s_{n},a_{n}\right),n=1,\ldots ,N\right\} \end{array}$$

where, if (s,a)∈D, *a* is assumed optimal in state *s*.

To address the two difficulties above, it is common to treat (2) as a constraint that the target reward function must verify, but select the latter so as to meet some additional regularization criterion, in an attempt to avoid the trivial solution (Ng et al., 2000). For the purpose of this work, we re-formulate IRL as


(3) $$\begin{array}{lll} \text{max} & 1^{⊤}v & \\ \text{ s.t.} & \left(p\left(s_{n},a_{n}\right)-p\left(s_{n},a^{\prime }\right)\right)v≽\varepsilon , & \forall \left(s_{n},a_{n}\right)\in D,a^{\prime }\in A,\\ & 0≼v-\gamma \underset{a\in A}{\text{ max }}{\text{P}}_{a}v≼R_{max}, & \end{array}$$

where ***p***(*s, a*) is the row-vector with element *s*′ given by **P**(*s*′∣*s, a*). In (3), we directly solve for *v*^π^ instead of r^*^, and then compute r^*^ as


$$\begin{array}{l} r^{*}=v-\gamma \underset{a\in A}{\text{ max }}{\text{P}}_{a}v. \end{array}$$

The IRL formulation in (3) implicitly assumes a reward *r* ≤ *R*_*max*_, which has no impact on the representative power of the solution. Moreover, it deals with the inherent ambiguity of IRL by maximizing the value of all states while imposing that the “optimal actions” are at least ε better than sub-optimal actions. The proposed formulation, while closely related to the simpler approaches of Ng et al. (2000), is simpler to solve and less restrictive in terms of assumptions.

It is worth noting that previous approaches on machine teaching in sequential tasks (Cakmak and Lopes, 2012; Brown and Niekum, 2019) assume (either implicitly or explicitly) that the IRL learners turn a demonstration into constraints that the reward function must verify, like those in (2). However, such constraints are built in a way that requires the learner to know (or, at least, be able to sample from) the teacher's policy π (Cakmak and Lopes, 2012; Brown and Niekum, 2019). As argued before, this is often inconvenient/unrealistic. Our formulation in (3) circumvents such limitation and has interest on its own. More efficient methods for IRL have been introduced (Balakrishnan et al., 2020) or considering differences in features between the teacher and the learner (Haug et al., 2018). Here, our focus is on the multiple learner aspect and so, for clarity, rely on simpler methods.

In the remainder of the paper, we refer to an “IRL agent” as defined by a rewardless MDP (S,A,P,γ) and such that, when given a demonstration D, outputs a reward r(D) obtained by solving (3). We write r^*^ to refer to the (unknown) target reward function, and v^*^ to denote the value function associated with π^*^(r^*^). Finally, unless if otherwise stated, all value functions are computed with respect to the MDP $\left(S,A,\text{P},r^{*},\gamma \right)$.

### 2.3. Machine Teaching in IRL

Let us now consider the problem of *teaching* an IRL agent. In particular, given an IRL agent, described by a rewardless MDP (S,A,P,γ), and a target reward function r^*^, we want to determine the “most concise” demonstration D such that r(D) is policy- equivalent to r^*^, i.e.,


$$\begin{array}{l} \pi^{*}\left(r^{*}\right)=\pi^{*}\left(r\left(D\right)\right). \end{array}$$

By “most concise,” we imply that there is a function, **effort**, that measures the teaching effort associated with any demonstration D (for instance, the number of examples in D). Teaching an IRL agent can thus be formulated as solving


(4) $$\begin{array}{ll} \underset{D}{\min} & \;\text{effort}\left(D\right)\\ \text{ s.t.} & \;\pi^{*}\left(r^{*}\right)=\pi^{*}\left(r\left(D\right)\right). \end{array}$$

The first approach to solving this problem was presented by Cakmak and Lopes (2012) using an incremental process. A more efficient approach was introduced by Brown and Niekum (2019), where the set of non-redundant demonstrations is found through the solution of a linear problem. The latter is the one used in this work. Note also that, in order to solve (4), the aforementioned approaches assume that the teacher knows r^*^—used to compute π^*^(r^*^)—and the learner model—used to compute r(D), π^*^(r^*^), and $\pi^{*}\left(r\left(D\right)\right)$. In the remainder of the paper, we also adhere to this assumption.

**Figure 1:**
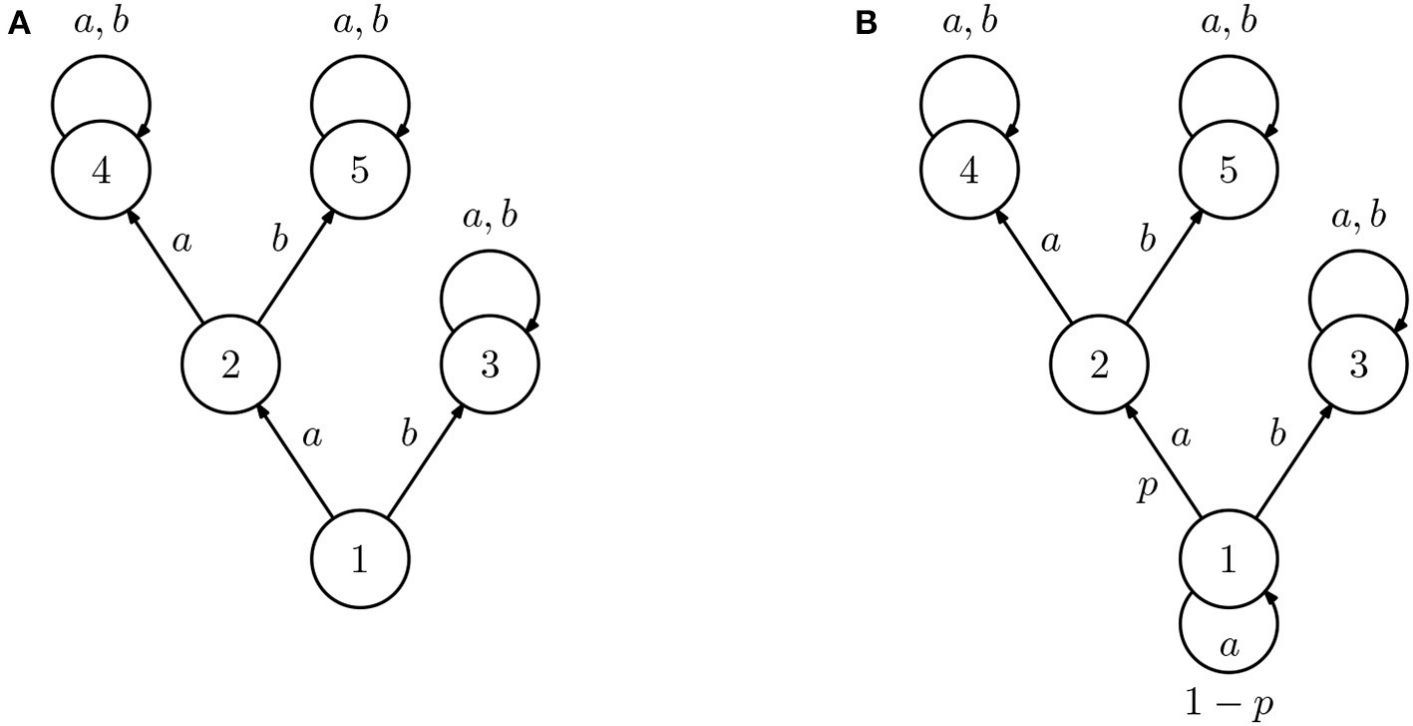
Diagram representing two inverse reinforcement learning (IRL) agents (all unmarked transition are deterministic). The two agents are similar in all states except 1, where action *a* always succeeds for agent **(A)** but only succeeds with probability *p* for agent **(B)**.

To illustrate the problem of machine teaching in IRL consider, for example, the IRL agent *A* in Figure 1A, defined as the rewardless MDP ({1, 2, 3, 4, 5}, {*a, b*}, **P**, γ), where the edges represent the transitions associated with the different actions (unmarked edges correspond to deterministic transitions) and γ > 0.5. If the target reward is


(5) $$\begin{array}{l} r^{*}={\left[\begin{array}{ccccc} 0, & 0, & 1, & 0, & 2 \end{array}\right]}^{⊤}, \end{array}$$

the optimal value function is given by


$$\begin{array}{l} v^{*}=\frac{1}{1-\gamma }{\left[\begin{array}{ccccc} 2\gamma^{2}, & 2\gamma , & 1, & 0, & 2 \end{array}\right]}^{⊤}, \end{array}$$

and the optimal policy selects action *a* in state 1 and action *b* in state 2, since 2γ > 1. Since both actions are equal in the remaining states, the most succinct demonstration should be, in this case,


$$\begin{array}{l} D=\left\{\left(1,a\right),\left(2,b\right)\right\}. \end{array}$$

As another example, consider the IRL agent *B* in Figure 1B. This learner is, in all aspects, similar to IRL agent *A* except that action *a* is now stochastic in state 1 and succeeds only with probability *p*. The optimal value function is now


$$\begin{array}{l} v^{*}=\frac{1}{1-\gamma }\left[\begin{array}{ccccc} u, & 2\gamma , & 1, & 0, & 2 \end{array}\right], \end{array}$$

where


$$\begin{array}{l} u=\text{ max }\left\{\frac{2\gamma^{2}p}{1-\gamma \left(1-p\right)},\gamma \right\}. \end{array}$$

Then, if


$$\begin{array}{l} p>\frac{1-\gamma }{\gamma }, \end{array}$$

the optimal policy is the same as in the previous case, as is the most concise demonstration. If, instead, the reverse inequality holds, the optimal policy is now to select action *b* in both states 1 and 2, and the best demonstration is


$$\begin{array}{l} D=\left\{\left(1,b\right),\left(2,b\right)\right\}. \end{array}$$

Finally, if *p* = (1 − γ)/γ, then both actions are equally good in state 1, and the most concise demonstration is just D={(2,b)}.

In the continuation, we extend the present setting, considering the situation where the teacher is faced with multiple inverse reinforcement learners.

## 3. Class-Teaching of Sequential Tasks

In this section, we present our first contributions. We formalize the problem of class-teaching multiple IRL agents. We then identify necessary conditions that ensure that we can teach all learners in a class simultaneously, i.e., using the same demonstrations for all. We finally provide a first algorithm that is able to teach under these conditions. We note that, although most examples in this section feature 2-learner settings, the conclusions hold for settings involving more than two agents, since the latter cannot be simpler than the former.

### 3.1. Teaching a Class of IRL Learners

Let us consider a teacher facing a heterogeneous class of *L* IRL agents, each one described as a rewardless MDP *M*_ℓ_^2^. We assume that the teacher perfectly knows the models *M*_1_, …, *M*_*L*_ and that the learners all adopt the IRL formulation in (3), given a demonstration D consisting of a set of state-action pairs.

Given a target reward function r^*^, the goal of the teacher is, once again, to find the “most concise” demonstration D that ensures that $r_{\ell }\left(D\right)$ is compatible with r^*^, where $r_{\ell }\left(D\right)$ is the reward computed by the IRL agent ℓ upon observing D. In other words, the goal of the teacher is to solve the optimization problem


(6) $$\begin{array}{ll} \underset{D}{\min} & \text{effort}\left(D\right)\\ \text{ s.t.} & \pi_{\ell }^{*}\left(r^{*}\right)=\pi_{\ell }^{*}\left(r_{\ell }\left(D\right)\right),\text{for }\ell =1,\ldots ,L\text{.} \end{array}$$

For the sake of concreteness, we henceforth consider effort(D)=|D|/|S|, roughly corresponding to the “percentage” of demonstrated states. The constraint in (6) states that the teacher should consider only demonstrations D that ensure the reward $r_{\ell }\left(D\right)$ recovered by each IRL agent ℓ is policy-equivalent to r^*^, for ℓ = 1, …, *L*.

In general, the problem (6) may not have a solution. In fact, there may be no single demonstration that ensures that all learners recover a reward function compatible with r^*^. Consider for instance a class comprising the agents *A* and *B* from Figure 1. Suppose that the target reward is the one in (5) and that


$$\begin{array}{l} p<\frac{1-\gamma }{\gamma }. \end{array}$$

If we provide the demonstration D={(2,b)}, the only constraint imposed by such demonstration is that *v*(5) ≥ *v*(4) + ε, which leads to the solution


$$\begin{array}{l} v=\frac{1}{1-\gamma }{\left[\begin{array}{ccccc} 1, & 1, & 1, & 1-\varepsilon \left(1-\gamma \right), & 1 \end{array}\right]}^{⊤}, \end{array}$$

corresponding to the reward


(7) $$\begin{array}{l} r={\left[\begin{array}{c} 1,1,1,1-\varepsilon \left(1-\gamma \right),1 \end{array}\right]}^{⊤}. \end{array}$$

Such reward does not verify the constraint in (6). For example, the policy that selects *a* and *b* in state 1 with equal probability is optimal with respect to the reward in (7), both for *A* and *B*. However, it is not optimal with respect to r^*^ for neither of the two. Repeating the derivations above for the demonstration D={(1,a),(2,b)}, we immediately see that the reward $r_{A}\left(D\right)$ will verify the constraint in (6) but not the reward $r_{B}\left(D\right)$. Conversely, if D={(1,b),(2,b)}, we immediately see that $r_{B}\left(D\right)$ will verify the constraint in (6) but not $r_{A}\left(D\right)$.

The example above brings to the forefront an immediate difficulty in teaching a group of heterogeneous IRL agents: since the relation between the reward and the policy tightly depends on the rewardless MDP describing the IRL agent, the ability of the teacher to ensure that an IRL agent recovers the desired reward/policy is strongly tied to the teacher's ability to provide a personalized demonstration. This is a fundamental difference from other MT settings, where the examples provided by the teacher directly encode the concept to be learned.

In the IRL case, the examples provided by the teacher (state-action pairs) provide only *indirect information* about the concept to be learned (the reward), the relation being greatly dependent on the particular learner considered. This observation is summarized in the following result, where we refer to a demonstration D as *complete* if there is a pair (s,a)∈D for every s∈S.

**Lemma 1**. *For two complete demonstrations*$D_{1}$*and*$D_{2}$*and two arbitrary IRL agents A and B*,


$$\begin{array}{l} \pi_{A}^{*}\left(r_{A}\left(D_{1}\right)\right)=\pi_{B}^{*}\left(r_{B}\left(D_{2}\right)\right) \end{array}$$

*only if*$D_{1}=D_{2}$.

*Proof*: By definition, a complete demonstration includes a state-action pair for every state s∈S with a corresponding optimal action. The constraints implied by the demonstration will necessarily lead both agents to learn similar policies. Conversely, if the agents learn different policies, either the demonstrations are different or incomplete.     □

In the continuation, we discuss in further detail how differences between learners affect the ability of the teacher to teach a whole class with a single demonstration.

### 3.2. Teaching Learners With Different Transition Probabilities

As argued before, assuming that the policy is provided to the learners in full (i.e., the demonstration is complete in the sense of Lemma 1) is often unrealistic. In the more natural situation of an incomplete demonstration, the conclusion of Lemma 1 no longer holds. To see why this is so, we first consider the case where the IRL learners differ only in their transition probabilities, i.e., each IRL learner is described as a rewardless MDP $M_{\ell }=\left(S,A,{\text{P}}_{\ell },\gamma \right),\ell =1,\ldots ,L$.

**Figure 2:**
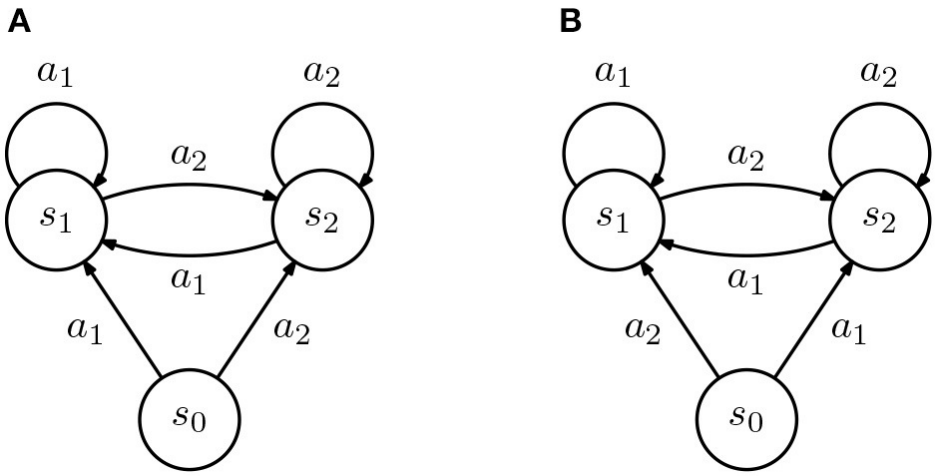
Diagram representing two inverse reinforcement learning (IRL) agents (all transition are deterministic). **(A,B)** The two agents are similar in all states except *s*_0_, where actions *a*_1_ and *a*_2_ lead to different states.

To aid in our discussion, we consider two simple IRL agents, depicted in Figure 2 and suppose that we provide a non-empty but incomplete demonstration to the agents. For concreteness, let $D=\left\{\left(s_{1},a_{1}\right)\right\}$. From our IRL formulation, we get the constraint that


$$\begin{array}{l} v\left(s_{1}\right)-v\left(s_{2}\right)\geq \varepsilon , \end{array}$$

for some ε > 0 which, by setting *R*_*max*_ = 1, leads to


$$\begin{array}{l} v\left(s_{0}\right)=v\left(s_{1}\right)=\frac{1}{1-\gamma };\;v\left(s_{2}\right)=\frac{1}{1-\gamma }-\varepsilon . \end{array}$$

Then, in state *s*_0_, both agents will necessarily recover a different policy. We state this observation in the following fact.

**Fact 1**. *Let*S*and*A*denote arbitrary finite state and action spaces, with*|S|>1*and*|A|>1, *and*D⊂S×A*a non-empty incomplete demonstration. Then, there exist two IRL agents*$\left(S,A,{\text{P}}_{A},\gamma \right)$*and*$\left(S,A,{\text{P}}_{B},\gamma \right)$*such that*


$$\begin{array}{l} \pi_{A}^{*}\left(r_{A}\left(D\right)\right)\neq \pi_{B}^{*}\left(r_{B}\left(D\right)\right). \end{array}$$

*In other words, an incomplete demonstration may lead to different policies in different agents*.

This is a negative result for class teaching: we show that the differences between the transition probabilities of the agents in a class may imply that the same reward leads to different optimal policies which, in turn, implies that there are cases where the same demonstration will lead to rewards that are not “compatible” with the target policy [i.e., do not verify the constraint in (6)]. This is particularly true for classes where the transition probabilities of the different learners exhibit large differences.

### 3.3. Teaching Learners With Different Discount Factors

We now address the case where the IRL learners differ in their discount, i.e., each IRL learner is described as a rewardless MDP $M_{\ell }=\left(S,A,\text{P},\gamma_{\ell }\right),\ell =1,\ldots ,L$. As we will see, this situation is similar to that discussed in section 3.2.

**Figure 3:**
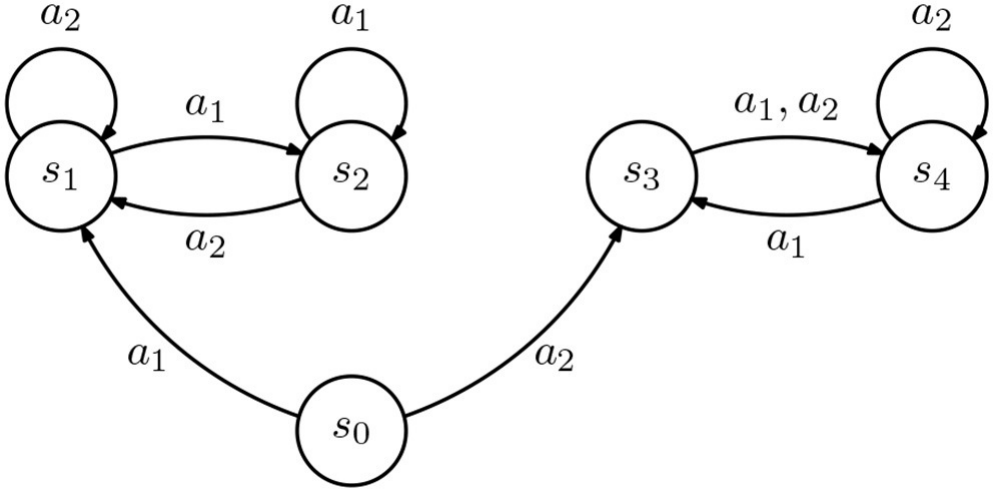
Diagram representing an inverse reinforcement learning (IRL) agent. All transitions occur with probability 1.

Consider two IRL agents, *A* and *B*, each described as a rewardless MDP $\left(S,A,\text{P},\gamma_{\ell }\right)$, where the transition probabilities are represented in Figure 3, and such that γ_*A*_ = 0.1 and γ_*B*_ = 0.9. Further, suppose that we provide the demonstration $D=\left\{\left(s_{2},a_{1}\right),\left(s_{4},a_{1}\right)\right\}$. From our IRL formulation, we get that


$$\begin{array}{l} v\left(s_{2}\right)-v\left(s_{1}\right)\geq \varepsilon ,\text{ and }v\left(s_{3}\right)-v\left(s_{4}\right)\geq \varepsilon , \end{array}$$

for some ε > 0.

Again setting *R*_*max*_ = 1, we get that


$$\begin{array}{l} v\left(s_{2}\right)=\frac{1}{1-\gamma };\;v\left(s_{1}\right)=\frac{1}{1-\gamma }-\varepsilon . \end{array}$$

Similarly, after some manipulation, we can conclude that *v*(*s*_4_) = *v*(*s*_3_) − ε,^3^ and


$$\begin{array}{l} v\left(s_{3}\right)=1+\gamma v\left(s_{4}\right)=1+\gamma \left(v\left(s_{3}\right)-\varepsilon \right). \end{array}$$

Solving for *v*(*s*_3_) yields


$$\begin{array}{l} v\left(s_{3}\right)=\frac{1}{1-\gamma }-\frac{\gamma }{1-\gamma }\varepsilon . \end{array}$$

Finally,


$$\begin{array}{l} v\left(s_{0}\right)=1+\gamma \text{ max }\left\{v\left(s_{1}\right),v\left(s_{3}\right)\right\}=\frac{1}{1-\gamma }-\gamma \varepsilon \text{ max }\left\{1,\frac{\gamma }{1-\gamma }\right\}. \end{array}$$

We can now conclude that, in state *s*_0_, both agents will recover a different policy, leading to the following fact.

**Fact 2**. *Let*S*and*A*denote arbitrary finite state and action spaces, with*|S|>4*and*|A|>1, *and*D⊂S×A*a non-empty incomplete demonstration. Then, there exist two IRL agents*$\left(S,A,\text{P},\gamma_{A}\right)$*and*$\left(S,A,\text{P},\gamma_{B}\right)$*such that*


$$\begin{array}{l} \pi_{A}^{*}\left(r_{A}\left(D\right)\right)\neq \pi_{B}^{*}\left(r_{B}\left(D\right)\right). \end{array}$$

*In other words, an incomplete demonstration may lead to different policies in different agents*.

It is interesting to note that the example above relies on more complex MDPs (i.e., with larger state-space), since the impact of the discount factor in the IRL agents only becomes noticeable if the agent is able to experience longer trajectories of states.

As in section 3.2, Fact 2 is a negative result for class teaching: we show that the differences between the discount factor of the agents in a class may imply that the same demonstration will lead to rewards that do not verify (6).

### 3.4. Teaching Learners With Different Reward Features

We conclude our analysis by considering the situation where the agents have different representations for the reward function. This situation is different than those considered before, since it does not concern differences in the IRL agent model (i.e., the rewardless MDP), but rather in the way the agents represent the reward.

In particular, if the two agents are represented by a common rewardless MDP (S,A,P,γ), from a common demonstration D both agents will recover the same value function *v* as a solution to (3). The difference between the two agents will thus be observed in the process of recovering the reward from *v*.

Suppose, then, that the IRL agents represent the reward as a linear combination of *K* features ϕ_*k*_, *k* = 1, …, *K*, i.e.,


$$\begin{array}{l} r_{w}\left(s\right)=\overset{K}{\underset{k=1}{\sum }}\phi_{k}\left(s\right)w_{k}=\phi^{⊤}\left(s\right)w, \end{array}$$

where ϕ_*k*_(*s*) is the value of the *k*th feature on state *s*, and **ϕ**(*s*) is a column-vector with *k*th component given by ϕ_*k*_(*s*). Then, given the value function *v*, we can compute, for example,


$$\begin{array}{l} \hat{r}=\text{argmin}∥v-\gamma \underset{a\in A}{\text{ max }}{\text{P}}_{a}v-r_{w}{∥}^{2}, \end{array}$$

where the minimization is over all possible ***r***_***w***_. In this case, the solution is the orthogonal projection of $v-{\text{ max }}_{a\in A}{\text{P}}_{a}v$ on the linear span of the set of features, i.e.,


$$\begin{array}{l} \hat{r}=\text{Proj}\left(v-\gamma \underset{a\in A}{\text{ max }}{\text{P}}_{a}v\right)\overset{\text{def}}{=}\Phi {\left(\Phi^{⊤}\Phi \right)}^{-1}\Phi^{⊤}\left(v-\gamma \underset{a\in A}{\text{ max }}{\text{P}}_{a}v\right). \end{array}$$

Let us then consider the case where the IRL learners differ in the set of features they use to represent the reward function. In particular, given two agents *A* and *B*, suppose that agent *A* uses features {ϕ_*A, k*_, *k* = 1, …, *K*}, and agent *B* uses a different set of features, {ϕ_*B, k*_, *k* = 1, …, *K*}^4^. If {ϕ_*A, k*_} and {ϕ_*B, k*_} both span the same linear space, both agents recover the same reward function and a single demonstration suffices to teach both agents the best possible reward. The two sets of features span the same subspace if there is an invertible *K* × *K* matrix **M** such that


$$\begin{array}{l} \Phi_{A}=\Phi_{B}\text{M}. \end{array}$$

In that case, given any reward function *r*,


$$\begin{array}{l} {\text{Proj}}_{A}\left(r\right)=\Phi_{A}({\Phi_{A}}^{⊤}\Phi_{A}{)}^{-1}\Phi_{A}^{⊤}r\\ \;=\Phi_{B}\text{M}({\text{M}}^{⊤}\Phi_{B}^{⊤}\Phi_{B}\text{M}{)}^{-1}{\text{M}}^{⊤}\Phi_{B}^{⊤}r\\ \;=\Phi_{B}\text{M}{\text{M}}^{-1}{\left(\Phi_{B}^{⊤}\Phi_{B}\right)}^{-1}{\text{M}}^{-⊤}{\text{M}}^{⊤}\Phi_{B}^{⊤}r\\ \;=\Phi_{B}{\left(\Phi_{B}^{⊤}\Phi_{B}\right)}^{-1}\Phi_{B}^{⊤}r\\ \;=\text{Pro}{\text{j}}_{B}\left(r\right). \end{array}$$

**Figure 4:**
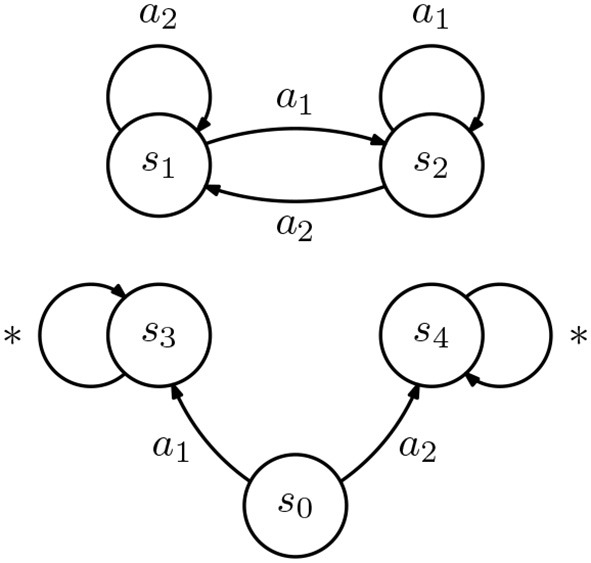
Diagram representing an inverse reinforcement learning (IRL) agent. All transitions occur with probability 1. Transitions labeled with “*” are associated with all actions a∈A.

However, if {ϕ_*A, k*_} and {ϕ_*B, k*_} span different spaces, an incomplete demonstration may lead to different policies in different agents. Consider two IRL agents, *A* and *B*, both described by the rewardless MDP in Figure 4. Assume that both consider the same discount γ, but agent *A* considers the reward features


$$\begin{array}{ll} \phi_{A,1} & ={\left[\begin{array}{ccccc} 0, & 1, & 0, & 0, & 1 \end{array}\right]}^{⊤},\;\phi_{A,2}={\left[\begin{array}{ccccc} 0, & 0, & 1, & 1, & 0 \end{array}\right]}^{⊤}, \end{array}$$

and agent *B* considers the reward features


$$\begin{array}{l} \phi_{B,1}=\left[\begin{array}{ccccc} 0, & 1, & 0, & 1, & 0 \end{array}\right],\;\phi_{B,2}=\left[\begin{array}{ccccc} 0, & 0, & 1, & 0, & 1 \end{array}\right]. \end{array}$$

Upon observing the demonstration $D=\left\{\left(s_{1},a_{1}\right)\right\}$, both agents will recover


$$\begin{array}{l} v=\frac{1}{1-\gamma }{\left[\begin{array}{ccccc} 1, & 1, & 1-\varepsilon \left(1-\gamma \right), & 1, & 1 \end{array}\right]}^{⊤}, \end{array}$$

and


$$\begin{array}{l} v-\gamma \underset{a\in A}{\text{ max }}{\text{P}}_{a}v={\left[\begin{array}{ccccc} 1, & 1, & 1-\varepsilon , & 1, & 1, \end{array}\right]}^{⊤}. \end{array}$$

It follows immediately that


$$\begin{array}{l} {\text{Proj}}_{A}\left(v-\gamma \underset{a\in A}{\text{ max }}{\text{P}}_{a}v\right)={\left[\begin{array}{ccccc} 0, & 1, & 1-\frac{\varepsilon }{2}, & 1-\frac{\varepsilon }{2}, & 1 \end{array}\right]}^{⊤},\\ {\text{Proj}}_{B}\left(v-\gamma \underset{a\in A}{\text{ max }}{\text{P}}_{a}v\right)={\left[\begin{array}{ccccc} 0, & 1, & 1-\frac{\varepsilon }{2}, & 1, & 1-\frac{\varepsilon }{2} \end{array}\right]}^{⊤}. \end{array}$$

The reward recovered by both agents *A* and *B* leads to a policy that matches the demonstration, but differs in the action selected by the agents in state *s*_0_. We thus get the following fact.

**Fact 3**. *Let*S*and*A*denote arbitrary finite state and action spaces, with*|S|>3*and*|A|>1, *and*D⊂S×A*a non-empty incomplete demonstration. Then, there exist two IRL agents A and B, both described by the same rewardless MDP*(S,A,P,γ)*but using different sets of reward features* {ϕ_*A, k*_} *and* {ϕ_*B, k*_}, *with different linear span, such that*


(8) $$\begin{array}{l} \pi_{A}^{*}\left(r_{A}\left(D\right)\right)\neq \pi_{B}^{*}\left(r_{B}\left(D\right)\right). \end{array}$$

*In other words, an incomplete demonstration may lead to different policies in different agents*.

Fact 3 is yet another negative result for class teaching: we show that the differences between the features that the learners in a class use to represent the reward function may imply that the same demonstration will lead to rewards that do not verify (6). We note, however, that the example leading to Fact 3 can be explained by the fact that the reward features of agents *A* and *B* do not span the whole space of possible rewards. If the reward features of both agents did span the whole space of possible rewards, we would be back in a situation where class teaching is possible.

The case where the space of reward features does not span the whole space of possible rewards, however, falls somewhat outside of our analysis, since it may not be possible at all for the IRL agents to recover a reward that is compatible with r^*^—i.e., even a single-agent setting, teaching may not be possible. For this reason, in the remainder of the paper, we assume that—even if different—the reward features used by the different agents always span the set of all possible rewards.

### 3.5. The Possibility of Class Teaching

We finally identify necessary conditions to ensure that two different learners (either in transition probabilities, discount, or reward features) recover reward functions compatible with r^*^ from a common demonstration D.

**Proposition 1**. *Given two IRL agents A and B which may differ in their transition probabilities, discount, and/or reward features, if*


$$\begin{array}{l} \pi_{A}^{*}\left(r^{*}\right)\neq \pi_{B}^{*}\left(r^{*}\right), \end{array}$$

*then, in general, the two IRL agents cannot be taught using a common demonstration*Dand recover a reward compatible withr^*^.

*Proof*: Let $s_{0}\in S$ be such that $\pi_{A}^{*}\left(s_{0};r^{*}\right)=a_{1}$ and $\pi_{B}^{*}\left(s_{0};r^{*}\right)=a_{2}$, with *a*_1_ ≠ *a*_2_, and suppose that we provide a common demonstration to agents *A* and *B*. Clearly, if either (*s*_0_, *a*_1_) or (*s*_0_, *a*_2_) appear in D, one of the agents will learn a reward that is not compatible with r^*^. On the other hand, if *s*_0_ does not appear in D, both agents will learn rewards according to which the policy that selects *a*_1_ and *a*_2_ with equal (and positive) probability is optimal, which are incompatible with r^*^.     □

Proposition 1 establishes that, in general, we cannot expect to achieve successful class teaching, where the same examples can be used by everyone. It also provides a verified way to test how different the learner can be before we need personalized teaching. The next corollary is a direct consequence of Proposition 1.

**Corollary 1 (Possibility of Class Teaching)**. If it is possible to class-teach a reward r^*^to two IRL agents A and B, *then*$\pi_{A}^{*}\left(r^{*}\right)=\pi_{B}^{*}\left(r^{*}\right)$.

Corollary 1 states the main challenge of class teaching in sequential tasks: if the differences between the different learners imply different optimal policies, they cannot be taught with a common demonstration.

## 4. Class Teaching Algorithms

In this section, we consider the implications of the results in section 3 in terms of the problem of optimally teaching sequential tasks to multiple learners. We first consider the problem of exact teaching and then move on to a more relaxed setting, where we allow the learners to learn the target task only approximately.

### 4.1. Exact Teaching

Let us first consider the problem of exact teaching. The goals of the teacher are twofold. First, it must ensure that all learners learn the correct task, i.e., have all students recover a reward such that the associated optimal policy (as computed by the student) is compatible with the target reward. Second, it must do so while optimizing the effort in teaching^5^.

The effort of providing a common demonstration to a class is independent of the number of learners in the class and, in that sense, the most efficient way to teach the class is to provide a single demonstration to the whole class. Unfortunately, in heterogeneous classes, it is unlikely that the conditions of Proposition 1 hold, so providing a single demonstration may lead students to learn an incorrect task.

Conversely, providing an individual demonstration to each learner ensures that all learners acquire the correct task, but it is the least efficient way of teaching, since the effort grows linearly with the number of students.

From the observations above, one very straightforward approach to class teaching is simply to provide a “class demonstration” containing all examples that are common across the class, and then complement this with individual demonstrations that make sure that the differences between the students are adequately addressed. In other words, we propose to combine class and personalized teaching. This simple idea is summarized in Algorithm 1, which we dub SplitTeach.

**Algorithm 1:** SplitTeach: Exact teaching IRL learners.

**Require:** IRL learners ℓ = 1, …, *L*
**Require:** Target reward r^*^
Compute $\pi_{\ell }^{*}\left(r^{*}\right)$ for ℓ = 1, …, *L*
For ℓ = 1, …, *L*, compute the demonstration $D_{\ell }$ necessary to determine $r_{\ell }\left(D_{\ell }\right)$ that is policy-equivalent to r^*^ (see section 2.3)
Compute $$\begin{array}{l} D_{\text{joint}}=\underset{\ell }{⋂}D_{\ell }; \end{array}$$
Provide $D_{\text{joint}}$ to all agents
**for** ℓ = 1, …, *L* **do**
Provide to each learner ℓ, the examples in $D_{\ell }-D_{\text{joint}}$
**end for**

SplitTeach extends the algorithm of Brown and Niekum (2019) to the class setting. The algorithm proceeds as follows: it identifies the optimal policy for each learner given the target reward r^*^. The teacher then demonstrates to the class those samples that are compatible across learners, and to each learner individually those samples that are specific to that learner's optimal policy^6^.

We can analyze the complexity of SplitTeach along several dimensions. Verifying if class teaching is possible or not implies comparing the optimal policies for the different learners. This comparison requires solving the MDP for each learner, which has a polynomial complexity.

On the other hand, computing which demonstrations to provide to each learner is linear in the number of learners and states. However, if we want to reduce the teaching effort by providing the most efficient demonstrations, we must identify which demonstrations introduce redundant constraints. This can be done through linear programming (Brown and Niekum, 2019), and requires solving as many linear programs as the size of the initial demonstrations set. In this case, since linear programming is solvable in polynomial time, we again obtain polynomial complexity.

Finally, we note that—with SplitTeach—each learner ℓ ends up observing


$$\begin{array}{l} D_{\text{joint}}\cup \left(D_{\ell }-D_{\text{joint}}\right)=D_{\ell }. \end{array}$$

However, the dataset D provided by the teacher has a number of examples given by


$$\begin{array}{l} |D|=|D_{\text{joint}}|+\overset{L}{\underset{\ell =1}{\sum }}|D_{\ell }-D_{\text{joint}}|, \end{array}$$

which corresponds to a saving in effort of $\left|D_{\text{joint}}|\times \left(L-1\right)/|S\right|$ when compared with individual teaching, for which


$$\begin{array}{l} |D|=\overset{L}{\underset{\ell =1}{\sum }}|D_{\ell }|. \end{array}$$

### 4.2. Approximate Teaching

In our discussion so far, we considered only exact demonstrations and investigated conditions under which all learners in the class are able to recover the desired reward function (or a policy-equivalent one) exactly. We could, however, consider situations where some error is acceptable.

#### Error in the Policy

As a first possibility, we could consider an extended setting that allows small errors in the reward recovered by (some of) the learners. In such setting, we could consider that, given ϵ > 0,


$$\begin{array}{l} \|\pi^{*}\left(r^{*}\right)-\pi^{*}\left(r\left(D\right)\right)\|<\epsilon , \end{array}$$

along the lines of Haug et al. (2018). Such approximate setting could allow reductions to the teaching effort in the case where class teaching is possible.

However, the impossibility results we established still hold even in the approximate case. In fact, when class teaching is not possible, we can find ϵ_*L*_ > 0 such that


$$\begin{array}{l} \|\pi^{*}\left(r^{*}\right)-\pi^{*}\left(r\left(D\right)\right)\|\geq \epsilon_{L}, \end{array}$$

i.e., we cannot reduce the error arbitrarily (for otherwise class teaching would be possible). The example in Figure 1 shows one such case, where the same demonstration, if provided to the two learners, would result in an error that could not be made arbitrarily small.

#### Loss in Value

Another alternative is to consider that we allow the learners to learn different rewards as long as the expected cumulative discounted reward is not far from the optimal.

Let us consider a scenario with two IRL agents, *A* and *B*, each one described as a rewardless MDP $\left(S,A,{\text{P}}_{\ell },\gamma \right)$, ℓ = *A, B*, differing only in the transition probabilities. Further assume that $\pi_{A}^{*}\left(r^{*}\right)\neq \pi_{B}^{*}\left(r^{*}\right)$, and suppose that we provide both learners with a complete demonstration D such that


$$\begin{array}{l} \pi_{A}^{*}\left(r_{A}\left(D\right)\right)=\pi_{A}^{*}\left(r^{*}\right). \end{array}$$

In other words, learner *A* is able to recover from D a reward that is policy equivalent to r^*^, i.e., such that


$$\begin{array}{l} v^{\pi_{A}^{*}\left(r_{A}\left(D\right)\right)}\left(s\right)=v^{*}\left(s\right) \end{array}$$

for all s∈S, where both functions $v^{\pi_{A}^{*}\left(r_{A}\left(D\right)\right)}$ and v^*^ are computed in the context of the MDP $\left(S,A,\text{P},r^{*},\gamma \right)$. Lemma 1 ensures that learner *B* will recover a reward $r_{B}\left(D\right)$ such that


$$\begin{array}{l} \pi_{B}^{*}\left(r_{B}\left(D\right)\right)=\pi_{A}^{*}\left(r_{A}\left(D\right)\right)\neq \pi_{B}^{*}\left(r^{*}\right). \end{array}$$

We can compute an upper bound to how much the performance of learner *B* strays from that of learner *A*.

For simplicity of notation, we henceforth write *v*^ℓ^ and **P**_ℓ_ to denote $v^{\pi_{\ell }^{*}\left(r_{\ell }\left(D\right)\right)}$ and ${\text{P}}_{\ell ,\pi_{\ell }^{*}\left(r_{\ell }\left(D\right)\right)}$, respectively, for ℓ = *A, B*. When we want to highlight the dependence of *v*^ℓ^ and **P**_ℓ_ on the demonstration D, we write $v^{\ell }\left(D\right)$ and ${\text{P}}_{\ell }\left(D\right)$ with the same meaning.

We have


(9) $$\begin{array}{l} v^{A}-v^{B}=r^{*}+\gamma {\text{P}}_{A}v^{A}-r^{*}-\gamma {\text{P}}_{B}v^{B}=\gamma ({\text{P}}_{A}v^{A}-{\text{P}}_{B}v^{B}). \end{array}$$

Some manipulation yields


$$\begin{array}{l} v^{A}-v^{B}=\frac{\gamma }{2}\left[\left({\text{P}}_{A}+{\text{P}}_{B}\right)\left(v^{A}-v^{B}\right)+\left({\text{P}}_{A}-{\text{P}}_{B}\right)\left(v^{A}+v^{B}\right)\right]. \end{array}$$

Defining


$$\begin{array}{ll} \overset{-}{\text{P}} & =\frac{1}{2}\left({\text{P}}_{A}+{\text{P}}_{B}\right)\;\overset{-}{v}=\frac{1}{2}\left(v^{A}+v^{B}\right), \end{array}$$

we get


$$\begin{array}{l} v^{A}-v^{B}=\gamma {\left(\text{I}-\gamma \overset{-}{\text{P}}\right)}^{-1}\left({\text{P}}_{A}-{\text{P}}_{B}\right)\overset{-}{v}. \end{array}$$

Noting that $\overset{-}{\text{P}}$ is still a stochastic matrix, the inverse above is well defined. Computing the norm on both sides, we finally get, after some shuffling,


(10) $$\begin{array}{l} {\left\|v^{A}-v^{B}\right\|}_{2}\leq \frac{\gamma }{1-\gamma }{\left\|{\text{P}}_{A}-{\text{P}}_{B}{\|}_{2}\|\overset{-}{v}\right\|}_{2}. \end{array}$$

As expected, the difference in performance between agents *A* and *B* grows with the difference between the corresponding transition probabilities.

Following similar computations as those leading to (10), we can now derive an approximate teaching algorithm, obtained by relaxing the requirement that every learner must recover a reward that is policy equivalent to the target reward. In particular, let us suppose that a demonstration D is provided to a heterogeneous class of *L* learners. Unlike the derivations above, we now admit that the learners may differ in their transition probabilities, discount, and reward features.

Given a target reward r^*^, let $v_{\ell }^{*}$ denote the corresponding optimal value function for learner ℓ, for ℓ = 1, …, *L*. On the other hand, when provided a demonstration D, each learner ℓ will recover a reward $r_{\ell }\left(D\right)$ and compute an associated policy $\pi_{\ell }^{*}\left(r_{\ell }\left(D\right)\right)$. As before, we write ${\text{P}}_{\ell }\left(D\right)$ to denote the associated transition probabilities. Then, the corresponding loss in performance of learner ℓ is given by


(11) $$\begin{array}{l} \text{Los}{\text{s}}_{\ell }\left(D\right)={\left\|v_{\ell }^{*}-{\left(\text{I}-\gamma_{\ell }{\text{P}}_{\ell }\left(D\right)\right)}^{-1}r^{*}\right\|}_{2}. \end{array}$$

Note that the loss in (11) is directly related to the bound in (10). In fact, when the demonstration that minimizes (11) matches that needed to teach one of the learners, the bound in (10) directly applies.

Assuming (as we have throughout the paper) that the teacher has a model of the learners, the value in (11) can be computed for every learner ℓ = 1, …, *L*. Therefore, the problem of the teacher can be reduced to the optimization problem


(12) $$\begin{array}{l} \underset{D}{\min}\;\overset{L}{\underset{\ell =1}{\sum }}{\left\|v_{\ell }^{*}-{\left(\text{I}-\gamma_{\ell }{\text{P}}_{\ell }\left(D\right)\right)}^{-1}r^{*}\right\|}_{2}^{2}. \end{array}$$

The optimization problem (12) can be solved using any suitable optimization method, such as gradient descent (Neu and Szepesvári, 2007). Unfortunately, the objective function is non-convex, meaning that optimization through local search methods can be stuck in local minima. Nevertheless, there are several good initialization that may mitigate the impact of local minima—e.g., we can use as initialization the optimal value function of one of the learners, or the average between the two. The resulting approach is summarized in Algorithm 2.

**Algorithm 2:** JointTeach: Approximate teaching multiple IRL learners.

**Require:** IRL learners ℓ = 1, …, *L*
**Require:** Target reward r^*^
Solve the optimization problem (12) to get D
Provide class demonstration D

We conclude by noting that, in this work, we considered only learners that, given a demonstration, are able to solve the corresponding IRL problem exactly. When this is not the case, and the learner is only able to solve the IRL problem approximately, available error bounds for such approximation could be integrated into JointTeach at the cost of a more complex optimization. Suppose that learner ℓ is able to compute only an approximate reward ${\hat{r}}_{\ell }\left(D\right)=r_{\ell }\left(D\right)+m_{\ell }$, where ||*m*_ℓ_|| = ε. Let ${\text{P}}_{\ell }\left(D,m_{\ell }\right)$ denote the transition probabilities associated with $\pi_{\ell }^{*}\left({\hat{r}}_{\ell }\left(D\right)\right)$. Then, (12) becomes


(13) $$\begin{array}{l} \underset{D}{\min}\;\overset{L}{\underset{\ell =1}{\sum }}\underset{m_{\ell }}{\text{ max }}{\left\|v_{\ell }^{*}-{\left(\text{I}-\gamma_{\ell }{\text{P}}_{\ell }\left(D,m_{\ell }\right)\right)}^{-1}r^{*}\right\|}_{2}^{2}. \end{array}$$

## 5. Simulations

In this section, we provide several illustrative examples^7^ of when class teaching can, or cannot, be made in different scenarios. We present two simple scenarios motivated by potential applications in human teaching, and two extra scenarios that show other possibilities of our algorithm, namely that it works in random MDPs, that they handle differences in terms of the discount γ, as well as more than 2 agents.

### 5.1. Scenarios

**Figure 5:**
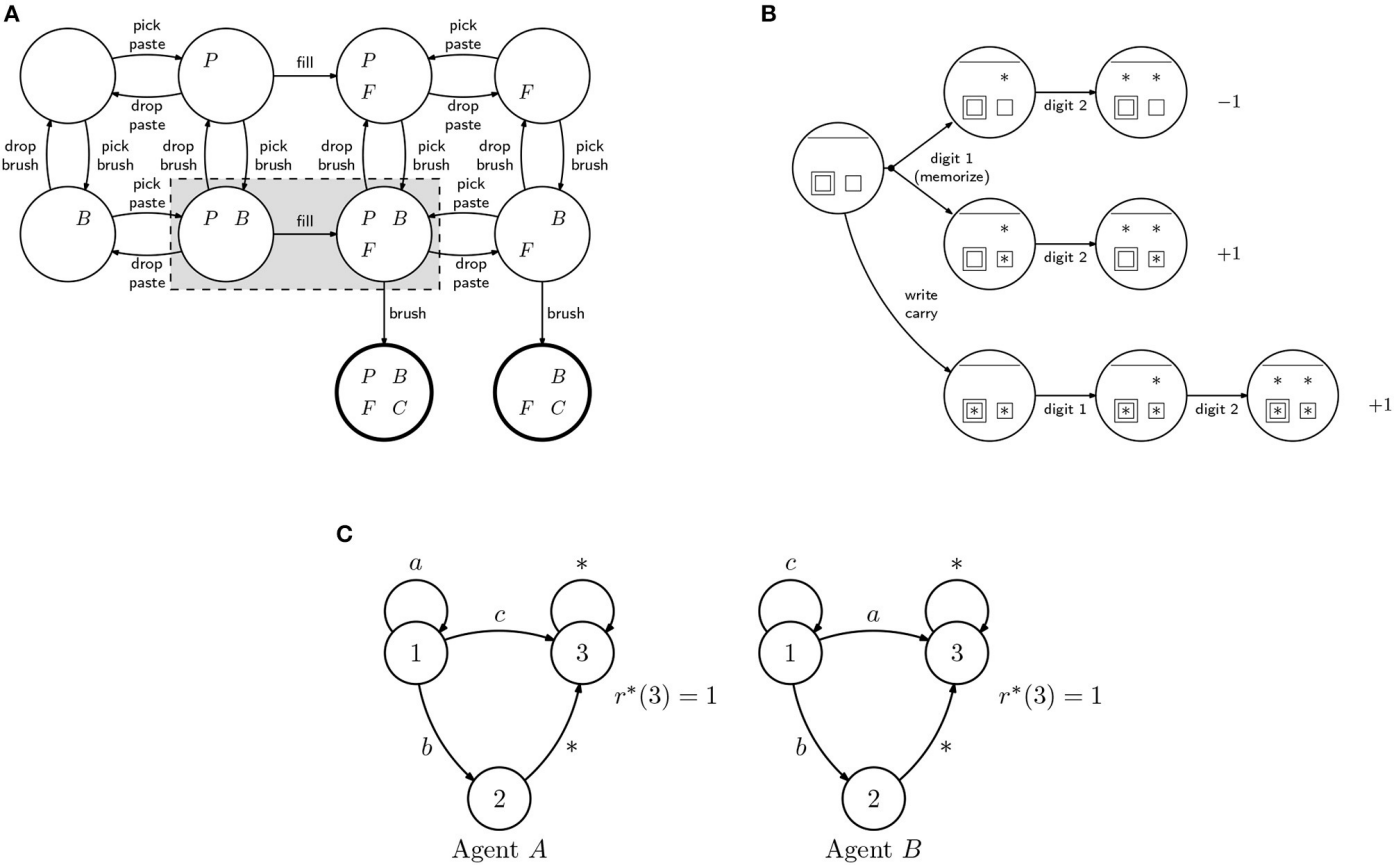
MDPs used in the examples. **(A)** Teeth brushing example: Each state is described by 4 binary features: *P* (holding paste), *B* (holding brush), *F* (brush with paste), and *C* (teeth clean). We consider the case where one user that cannot hold two objects at the same time and so some of the states are inaccessible (states in the shaded region). **(B)** Addition with carry: Simple model of 2-digit addition with a single carry. The asterisks represent digits. Some learners are able to memorize the carry digit (the single square) and do not have to explicit write it (the double square). If memorizing may fail (top path), the teacher might suggest to write the carry digit explicitly (lower path). Without writing the carry digit, a learner with difficulties may forget it (the top branch has higher probability), obtaining the wrong result. **(C)** Alternative paths MDP: 3-state MDP, where the task to be learned is to reach state 3, for which there are two paths: a direct one and a longer one, going through state 2. The two agents differ in the transition probabilities associated with actions *a* and *c*.

**Scenario 1. Brushing teeth (cognitive training):** Training sequential tasks is very important for many real-world applications. For instance, elderly whose cognitive skills are diminishing often struggle to plan simple tasks such as brushing their teeth or dressing up (Si et al., 2007). Motivated by such situations, we model the problem of training a group of learners in the different steps required to brush their teeth (Figure 5A). To brush the teeth, the brush (*B*) and toothpaste (*P*) must be picked; the brush must be filled (*F*) with toothpaste; only then brushing will lead to clean teeth (*C*). People may forget to put the paste, or may have coordination problems and be unable to hold the brush while placing the paste.

**Scenario 2. Addition with carry (education):** When teaching mathematical operations, teachers need to choose among different algorithms to perform those operations, taking into account the level of the learners, their capabilities for mental operations, and how much practice they had (Putnam, 1987). Let us consider addition with carry. For some learners, it might be useful to write down the carry digit to avoid confusion. A more advanced learner might find it confusing or even boring to be forced to make such auxiliary step. We can model this problem as the MDP in Figure 5B. The asterisks indicate which of the digits of the result have been computed (top). The square indicates whether or not the carry digit is memorized, while the double square indicates whether or not the carry digit is written down. A learner with bad memory may prefer to write down the carry digit; there is a larger probability of forgetting it and getting a wrong result.

**Scenario 3. Random MDPs:** To further illustrate the application of our approach in a more abstract scenario (ensuring that our algorithm is not exploiting any particular structure of the previous scenarios), we also consider randomly generated MDPs with multiple states (5–20 states), actions (3–5 actions), and rewards. The transition probabilities and reward are sampled from a uniform distribution.

**Scenario 4. Difference in discount factor** γ**:** In a fourth scenario, we consider two IRL learners described by the rewardless MDP depicted in Figure 1, but where γ_*A*_ = 0.9 and γ_*B*_ = 0.01, respectively. In this case, one learner is more “myopic” (i.e., eager to receive a reward) than the other. The policy in state 1 is different, so class teaching in not possible.

**Scenario 5. Alternative MDP:** In a fifth scenario, we consider two IRL learners, *A* and *B*, described by the alternative (rewardless) MDPs in Figure 5C. In this case, the task to be learned is to reach state 3, but the two agents differ in the transition probabilities associated with actions *a* and *c*. For agent *A*, *c* is the best action; for agent *B*, *a* is the best action.

**Scenario 6. 3 agents:** In a final scenario, we consider a scenario involving 3 learners. The scenario is mostly the same as Scenario 1, but we now consider 2 constrained agents and 1 non-constrained agent. We use Algorithm 1, where we first identify the examples than can be presented to all agents simultaneously, and then consider the agents one by one.

### 5.2. Methodology

We now describe in greater detail the methodology used to evaluate our algorithms in simulation.

Most of our scenarios consider two different agents, dubbed *A* and *B*, the single exception being Scenario 6, where we consider 3 agents, two of which are similar (see section 5.1). We evaluate the performance of our algorithms in terms of *effort* and *error in the value function*. Effort measures the percentage of states for which the teacher must provide demonstration (i.e., the number of samples in D) in relation to size of the state-space, as defined in page 6. Specifically,


(14) $$\begin{array}{l} \text{effort}\left(D\right)=\frac{\left|D\right|}{\left|S\right|}. \end{array}$$

The error in the value function is the average difference between the value of the policy estimated by the different agents and that of the optimal policy, i.e.,


(15) $$\begin{array}{l} \text{error}\left(D\right)=\frac{1}{L}\overset{L}{\underset{\ell =1}{\sum }}\frac{1}{\left|S\right|}\underset{s\in S}{\sum }\left(v^{\pi_{\ell }^{*}\left(r_{\ell }\left(D\right)\right)}\left(s\right)-v^{*}\left(s\right)\right). \end{array}$$

To provide a basis for comparing the performance of our proposed algorithms, use three baselines:

- *Individual*, where we teach each agent individually, i.e., we provide an individual demonstration $D_{\ell }$ for each agent ℓ, ℓ = *A, B*. In this case, the teacher provides a total of
  $$\begin{array}{l} \left|D|=\overset{L}{\underset{\ell =1}{\sum }}|D_{\ell }\right| \end{array}$$
  examples. Hence, we expect this baseline to provide an upper bound on the teaching effort, as each learner receives a different demonstration. Conversely, we expect this baseline to provide a lower bound on the error, as each learner receives the best demonstration possible;
- *Class* ℓ, with ℓ = *A, B*, where we provide both learners with a common demonstration, computed as if all agents were equal to agent ℓ. In this case, the teacher provides a total of
  $$\begin{array}{l} \left|D|=|D_{\ell }\right| \end{array}$$
  examples. This demonstration should provide a lower bound in terms of effort, but will potentially incur in errors, since the it does not take into consideration the difference between the learners.

The simulation results are collected through the following procedure:

1. We compute $\pi_{\ell }^{*}\left(r^{*}\right)$, for all agents ℓ;
2. Verify the conditions of Proposition 1;
  - if class teaching is possible, set $D=D_{A}$, or $D=D_{B}$;
  - otherwise, for SplitTeach, compute D as described in Algorithm 1;
  - for JointTeach, computed D as described in Algorithm 2;
3. provide D to the different agents, according to the aforementioned procedure;
4. for each agent ℓ, compute $r_{\ell }\left(D_{\ell }\right)$ using (3);
5. for each agent ℓ, compute $\pi_{\ell }^{*}\left(r_{\ell }\left(D\right)\right)$ using value iteration;
6. compute the effort using (14);
7. compute the error using (15).

Due to the stochasticity of the different environments, the results reported correspond to the averages over 100 independent runs of each scenario.

### 5.3. Results

Table 2 presents the results on the six scenarios described in section 5.1. To the extent of our knowledge, our work is the first to address machine teaching to multiple IRL learners. For this reason, we compare our algorithm with two natural baselines: teaching each agent individually (appearing in the table as “Individual”), where each agent gets an individualized demonstration, and teaching the whole class ignoring the differences between agents—by considering all agents to be like agent ℓ, ℓ = *A, B* (appearing in the table either as “Class *A*” or “Class *B*”). As discussed above, we expect individual teaching to provide the best results in terms of task performance across the class (shown in the columns marked with “$\overset{-}{v}$”), but at a greater cost in terms of effort (shown in the columns marked with “effort”). On the other hand, we expect the baselines that ignore the differences between agents (“blind” class teachers) to provide the best results in terms of effort, but often at a cost in task performance.

**Table 2:** Results for class teaching in five different Markov decision problems (MDPs).

	**1. Brushing**		**2. Addition**		**3. Random**	
	**effort**	*v* − v^*^	**effort**	*v* − v^*^	**effort**	*v* − v^*^
Individual	0.5	0.0	1.3	0.0	1.0	0.0
Class *A*	0.3	–3.9	0.6	–0.4	0.5	–4.1
Class *B*	0.3	–17.0	0.6	–3.2	0.5	–4.4
SplitTeach	0.4	0.0	0.7	0.0	0.7	0.0
JointTeach	0.3	–2.2	0.6	–0.4	0.5	–2.0
	**4. Different** γ		**5. Alternative**		**6. 3 Agents**	
	**effort**	*v* − v^*^	**effort**	*v* − v^*^	**effort**	*v* − v^*^
Individual	0.8	0.0	0.3	0.0	0.7	0.0
Class *A*	0.4	0.0	0.2	–4.5	0.3	–5.2
Class *B*	0.4	–3.6	0.2	–4.5	0.3	–11.0
SplitTeach	0.6	0.0	0.3	0.0	0.4	0.0
JointTeach	0.4	0.0	0.2	–0.9	0.3	–3.0

*We present the total effort and the average difference for all states of the value function of the learned policy and the value function for the optimal policy. We consider five conditions: individual teaching, teaching as if the class was homogeneous (Class A and Class B) and both our approaches (SplitTeach and JointTeach)*.

As can be seen in the results, our algorithms are able to strike a balance between these two extreme approaches to different extents. Specifically,

- In the Brushing scenario, neither “blind” class teaching approaches could teach the task, even if the effort was lower. Individual teaching could teach the task, but with maximum effort. SplitTeach could reduce the effort while still guaranteeing teaching, while JointTeach ended up converging to a “blind” class teaching strategy.
- In the addition scenario and the scenario featuring different discounts, the results are similar. One remarkable difference is that, in these two scenarios, SplitTeach was not able to save in effort at all, when compared with the individual teaching.
- When considering random MDPs, the advantages of our approaches become clearer: SplitTeach is significantly more efficient than individual teaching, while still attaining perfect performance. Conversely, *JointTeach* is the most efficient approach, at a minimal loss in performance.
- In the scenario with the alternative 3-state MDP, SplitTeach showcases maximum effort, while JointTeach showcases minimum effort. The performance of the latter is significantly better than “blind” class teaching, even if not optimal.

## 6. Conclusions

In this work, we formalized the problem of class teaching for IRL learners, studied its properties, and introduced two algorithms to address this problem. We identified a set of conditions that determine whether class teaching is possible or not. Contrary to several recent results for density estimation and supervised learning (Zhu et al., 2017; Yeo et al., 2019), where class teaching is always possible (even if with some added effort), in the case of IRL teaching, our results establish that class teaching is not always possible.

We illustrated the main findings of our paper by comparing our proposed algorithms in several different simulation scenarios with natural baselines. Our simulation results confirmed that our class teaching approaches are often able to teach as well as individual teaching, and often with a lower effort. The results in this work provide a quantitative evaluation of when class teaching is possible. As a side contribution, we showed also a simpler way to solve the IRL problem using directly the value function.

In this work, our presentation focused almost exclusively on scenarios with only two agents. However, we showed in the results that a trivial extension to more agents can be made by considering first class teaching, and then individual teaching. A more efficient method could consider the creation of a class partition, as presented in the work of Zhu et al. (2017).

One assumption we make throughout the paper is that the teacher knows the model of the students exactly. In a practical situation, without any added information/interaction, this assumption might be unrealistic. We could instead consider that the teacher does not have the exact model, but a distribution describing the student variation. In this case, the distribution could be used to decide when to do individual teaching or group teaching. The group teaching might still be possible, but some form of interaction might be needed (Walsh and Goschin, 2012; Haug et al., 2018; Melo et al., 2018).

We can envision several applications of this work in the teaching of humans. For applications involving humans, the complexity of the algorithm is not a problem, but the problem is assumption of knowing the learner's decision-making process (i.e., the rewardless MDP describing the human). In future work, we will consider how to include interaction in the teaching process, to overcome the lack of knowledge regarding the human learner, as was done for other teaching problems (Melo et al., 2018). Other applications of machine teaching include the study of possible attacks to machine learners (Mei and Zhu, 2015). We can use our approach to see if a set of learners can be attacked simultaneously or not.

## Data Availability Statement

Publicly available datasets were analyzed in this study. This data can be found at: https://github.com/maclopes/learnandteachinIRL.

## Author Contributions

All authors listed have made a substantial, direct and intellectual contribution to the work, and approved it for publication.

## Funding

This work was supported by national funds through the Portuguese Fundação para a Ciência e a Tecnologia, through Grant UID/CEC/50021/2020 and the HOTSPOT project, with reference PTDC/CCI-COM/7203/2020; and INFOCOS PTDC/CCI-COM/32378/2017. It was also funded by the EU H2020 through the RIA project iV4xr : 856716, and by the Air Force Office of Scientific Research under award number FA9550-19-1-0020.

## Conflict of Interest

The authors declare that the research was conducted in the absence of any commercial or financial relationships that could be construed as a potential conflict of interest.

## Publisher's Note

All claims expressed in this article are solely those of the authors and do not necessarily represent those of their affiliated organizations, or those of the publisher, the editors and the reviewers. Any product that may be evaluated in this article, or claim that may be made by its manufacturer, is not guaranteed or endorsed by the publisher.

## Footnotes

^1^This happens, for example, if *r* − *r*′ is a *potential function* (Ng et al., 1999).

^2^Note that we allow different learners to have different models.

^3^From our IRL formulation, it follows that *v*(*s*_4_) = min{*v*(*s*_3_) − ε, *R*_*max*_ + γ*v*(*s*_3_)}. However, if ε > 0 and *R*_*max*_ > 0, we have that *v*(*s*_3_) − ε < *R*_*max*_ + γ*v*(*s*_3_).

^4^We focus on the benign case in which both agents consider the same number of features. The negative results we present trivially hold if that is not the case.

^5^Recall that we consider the effort to depend directly on the number of examples provided.

^6^The algorithm could be further improved, considering subgroups that can minimize the teaching effort and providing samples to these subgroups. Such approach would bring additional savings in effort, but would require the computation of such subgroups, which is a combinatorial problem and adds to the complexity of the algorithm. We do not consider such variation in this paper.

^7^Code for some of the simulations is available at https://github.com/maclopes/learnandteachinIRL.
